# Effect of medroxyprogesterone acetate dose in progestin-primed ovarian stimulation on pregnancy outcomes in poor ovarian response patients with different body mass index levels

**DOI:** 10.3389/fendo.2024.1352522

**Published:** 2024-04-18

**Authors:** Qianjie Zhang, Shaojing He, Yicen Meng, Tailang Yin, Lei Ming, Jing Yang, Saijiao Li

**Affiliations:** Reproductive Medical Center, Renmin Hospital of Wuhan University, Hubei Clinical Research Center for Assisted Fertility and Embryo Development, Wuhan, China

**Keywords:** progestin-primed ovarian stimulation, medroxyprogesterone acetate, POSEIDON criteria, body mass index, pregnancy outcome

## Abstract

**Background:**

For the poor ovarian response (POR) population, the relationship between medroxyprogesterone acetate (MPA) dose in progestin-primed ovarian stimulation (PPOS) and clinical outcome is still unclear. This study aims to explore the effect of MPA dose in PPOS on clinical outcomes in POSEIDON group 3 and 4 patients with different body mass index (BMI) levels, hoping to provide clinical doctors with better options for controlled ovarian hyperstimulation (COH) programs.

**Methods:**

This is a retrospective analysis of 253 oocyte retrieval cycles of POSEIDON group 3 and 4 patients who underwent PPOS protocol in IVF/ICSI treatment at the Reproductive Medical Center of Renmin Hospital of Wuhan University from March 2019 to April 2022. The effects of different MPA doses (8 mg/d or 10 mg/d) on pregnancy outcomes were compared in normal BMI (18.5–24 kg/m^2^) and high BMI (≥24 kg/m^2^) patients, and multivariate logistic regression analysis was performed to analyze the factors affecting pregnancy outcomes.

**Results:**

For normal BMI patients, the 8-mg/d MPA group had a higher embryo implantation rate (33.78% vs. 18.97%, *P* = 0.012). For high BMI patients, the 10-mg/d MPA group had a higher HCG positive rate (55.00% vs. 25.00%, *P* = 0.028), clinical pregnancy rate (50.00% vs. 20.00%, *P* = 0.025), and cumulative pregnancy rate (37.74% vs. 13.79%, *P* = 0.023) compared with the 8-mg/d MPA group. There was no significant difference in cumulative live birth rate between the 8-mg/d and 10-mg/d MPA groups in patients with normal or high BMI. The results of multivariate logistic regression showed a significant correlation between MPA dose and cumulative pregnancy in the high BMI population (OR = 0.199, 95% CI: 0.046~0.861, *P* = 0.031).

**Conclusions:**

For POR patients with high BMI, 10 mg/d of MPA in the PPOS protocol had a higher cumulative pregnancy rate than 8 mg/d of MPA, but it had no significant effect on the cumulative live birth rate.

## 1 Introduction

With the increased number of late marriage and childbearing couples, poor ovarian response (POR) patients receiving assisted reproductive technology (ART) fertility treatment have also increased. POR refers to a pathological state in which the ovaries respond poorly to exogenous gonadotropin stimulation in patients receiving ART, which is characterized by an increased cycle cancellation rate and decreased number of oocytes and clinical pregnancy rate (1). It has been reported that more than 30% of women can be diagnosed with POR during controlled ovarian hyperstimulation (COH) (2). Therefore, the diagnosis and treatment of POR patients need to be given more attention.

Currently, many ovarian stimulation regimens have been proposed to improve the prognosis of POR patients. For example, progestin-primed ovarian stimulation (PPOS) proposed in recent years can effectively suppress the premature luteinizing hormone (LH) surge and increase the number of oocytes retrieved by using the exogenous progesterone medroxyprogesterone acetate (MPA) and has gradually been widely used in POR patients (3–6). The commonly used dose of MPA in the PPOS protocol is 10 mg/d, and the lowest dose that can suppress the premature LH surge is 4 mg/d (7). Previous studies have found that in a population with normal ovarian reserve, the dose of MPA is related to clinical outcome, and the use of higher doses of MPA in patients with high body mass index (BMI) is beneficial for achieving higher embryo implantation rates, clinical pregnancy rates, and live birth rates. However, a high dose of MPA may have deep pituitary suppression, thus increasing the total dose and duration of gonadotropin (8, 9). Whether MPA has a dose-dependent effect on IVF/ICSI outcomes and is influenced by BMI has attracted the attention of researchers. However, there is no literature on the relationship between MPA dose and IVF/ICSI pregnancy outcomes in POR patients with different BMI levels according to the POSEIDON criteria. Therefore, we conducted a retrospective study to investigate the effect of different MPA doses (8 mg/d and 10 mg/d) in the PPOS protocol on the clinical outcomes of POSEIDON group 3 and 4 patients with different BMI levels and whether using smaller doses of MPA (8 mg/d) is more beneficial for clinical outcomes.

## 2 Materials and methods

### 2.1 Study design and patients

This was a retrospective study of POR patients who underwent PPOS protocol in IVF/ICSI treatment at the Reproductive Medical Center of Renmin Hospital of Wuhan University from March 2019 to April 2022. This study was approved by the Ethics Committee of Renmin Hospital of Wuhan University (WDRY2018-K009) and accorded with the basic principles of the Helsinki Declaration.

In this study, we included POSEIDON group 3 and 4 patients. According to the POSEIDON criteria (10), POSEIDON group 3 is defined as female age <35 years, antral follicle count (AFC) <5, and/or anti-Müllerian hormone (AMH) <1.2 ng/ml, and POSEIDON group 4 is defined as female age ≥35 years, AFC <5, and/or AMH < 1.2 ng/ml. The exclusion criteria included patients >45 years (11), uterine malformation, polycystic ovary syndrome, endometrial disease, severe endometriosis, chromosomal abnormality, recurrent miscarriage, and endocrine diseases (thyroid dysfunction, hyperprolactinemia, diabetes). The cycle of mixed transfer of embryos obtained from other cycles is also excluded.

A total of 253 oocyte retrieval cycles were included according to the above inclusion and exclusion criteria. According to the China BMI classification (12, 13), patients were divided into two categories: normal BMI (18.5–24 kg/m^2^) and high BMI (≥24 kg/m^2^). The two categories of patients were further divided into the 8-mg/d MPA group and the 10-mg/d MPA group.

### 2.2 Clinical setting

All patients received the PPOS protocol. Based on previously published articles (5), patients were orally administered 8 mg/d or 10 mg/d of MPA (Beijing ZhongXin Pharmaceutical, China) and injected with 150–300 IU/d of human menopausal gonadotrophin (hMG, Livzon, China) or recombinant human follitropin (Gonal-F, Merck, Switzerland) from menstrual cycle day 2 or 3 until the trigger day. Gonadotropin dose was adjusted every 2 to 3 days after assessing follicle development based on transvaginal ultrasound and blood hormone levels. Final follicle maturation was triggered with 10,000 IU of human chorionic gonadotropin (hCG, Livzon, China) when at least one follicle reached the diameter of 18 mm or two follicles reached the diameter of 17 mm. After 34 to 36 h, the oocytes with diameter ≥12 mm were retrieved under the guidance of transvaginal ultrasound. The oocytes were fertilized by conventional IVF/ICSI according to semen parameters.

Fertilization was assessed 16 h to 18 h after oocyte retrieval. Seventy-two hours after oocyte retrieval, the cleavage status and fragmentation ratio of embryos were observed. Cleavage embryos were scored and graded as follows: grade I (uniform blastomere size, no fragmentation), grade II (uniform blastomere size, fragmentation <20%), grade III (uneven blastomere size, fragmentation <50%), and grade III (uneven blastomere size, fragmentation >50%). High-quality embryos were defined as blastomeres with six to eight cells and fragmentation <20%; transferable embryos were defined as blastomeres with six or more cells and embryo grade III or higher. Blastocyst culture was performed according to embryo quality and the patient’s wishes. The blastocyst was graded according to the Gardner scoring standard (14). Due to the effect of high progesterone levels on endometrial receptivity, all embryos obtained were frozen.

### 2.3 Frozen-thawed embryo transfer and follow-up

Frozen embryo transfer (FET) was performed 2 months after oocyte retrieval. Patients with regular menstrual cycles underwent natural cycles, and one to two cleavage embryos or blastocysts were transferred 3 or 5 days after ovulation. Patients with irregular menstruation underwent hormone replacement therapy (HRT); 3–6 mg/d of estradiol valerate tablets (Progynova, Bayer, Germany) were administered from day 3 of the menstrual cycle, and when the endometrial thickness ≥8 mm, 40 mg/d of progesterone and 30 mg/d of dydrogesterone were administered orally. For patients using gonadotropin-releasing hormone agonist (GnRH-a) downregulation combined with hormone replacement therapy (GnRH-a + HRT), 3.75 mg of GnRH-a (leuprorelin acetate, China) was given subcutaneously during menstruation, and the HRT was started 30 days later to prepare the endometrium. Luteal support was maintained until 10–12 days after transplantation. Serum β-HCG was detected 10–12 days after transplantation, and β-HCG >10 IU/L was defined as HCG positive. Clinical pregnancy was defined as gestational sac observed by vaginal ultrasonography 30 days after transplantation; pregnancy loss that occurred before 12 weeks was defined as early miscarriage; live birth after 28 weeks of gestation was defined as live birth. The deadline for follow-up of the included patients was August 2023.

### 2.4 Outcome measures and definition

The primary outcomes included cumulative pregnancy rate and cumulative live birth rate (CLBR) per oocyte retrieval cycle. The secondary outcomes included the number of oocytes retrieved, the number of mature oocytes, mature oocyte rate, 2PN fertilization rate and cleavage rate, high-quality embryo rate, the number of transferable embryos, cycle cancellation rate, HCG positive rate, embryo implantation rate, clinical pregnancy rate, and early miscarriage rate.

When there were no oocytes retrieved or no transferable embryos, the cycle was canceled. Cumulative pregnancy rate per oocyte retrieval cycle was defined as the ratio of the number of clinical pregnancy after all embryos have been transferred to the total number of oocyte retrieval cycles. CLBR was defined as the ratio of the number of live births after all embryos have been transferred to the total number of oocyte retrieval cycles (15).

### 2.5 Statistical analysis

SPSS 26.0 (IBM Corp., USA) was used for analysis. Measurement data conforming to a normal distribution were expressed as mean ± SD, and the independent sample *t*-test was used to compare variables between groups. Enumeration data were expressed as frequency (%). Categorical variables were compared using the chi-square test or Fisher’s precision probability test. Multivariate logistic regression was used to analyze the relationship between various factors and pregnancy outcome, and the odds ratio (OR) with 95% confidence intervals (CI) was calculated. Statistical significance was defined as *P*-value <0.05.

## 3 Results

**Figure 1:**
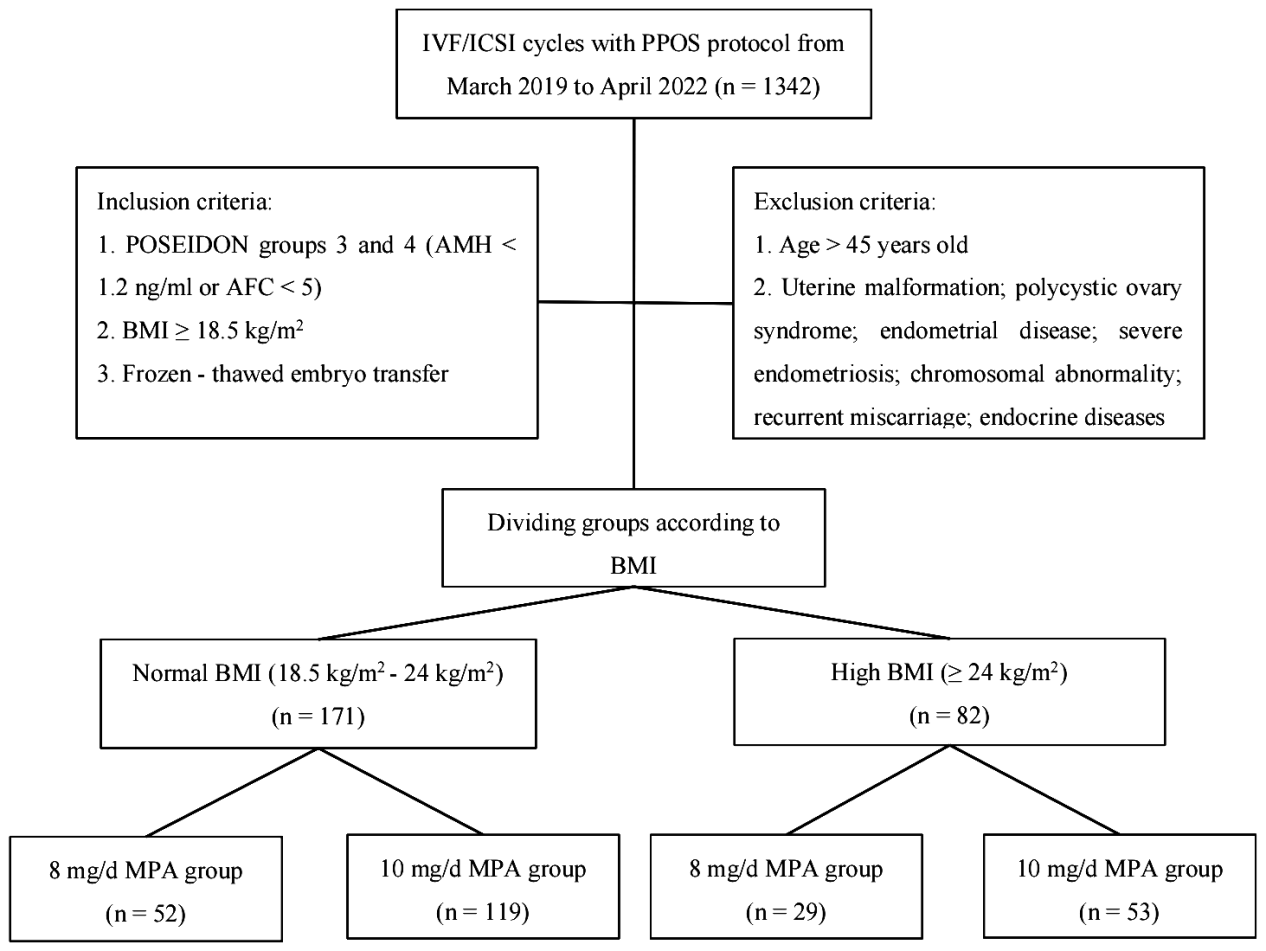
Flowchart of this study.

The research flowchart is shown in **Figure 1**. A total of 253 oocyte retrieval cycles were included in this study, with 171 oocyte retrieval cycles for normal BMI patients (18.5–24 kg/m^2^) and 82 oocyte retrieval cycles for high BMI patients (BMI ≥24 kg/m^2^). The baseline characteristics of the different MPA dose groups in normal BMI and high BMI patients are shown in **Table 1** and indicate that there were no significant differences in these indicators.

**Table 1:** Baseline characteristics of the different MPA dose groups.

Variable	Normal BMI			High BMI			
	8-mg/d MPA group (*n* = 52 cycles)	10-mg/d MPA group (*n* = 119 cycles)	*P-*value	8-mg/d MPA group (*n* = 29 cycles)	10-mg/d MPA group (*n* = 53 cycles)	*P-*value	
Age (years)	34.90 ± 5.23	34.62 ± 4.21	0.709	35.48 ± 4.72	34.32 ± 4.94	0.304	
BMI (kg/m^2^)	21.35 ± 1.64	21.27 ± 1.56	0.761	26.38 ± 1.30	25.95 ± 1.86	0.273	
Duration of infertility (years)	4.27 ± 4.17	4.25 ± 3.12	0.976	5.60 ± 5.31	4.74 ± 4.13	0.419	
Basal FSH (IU/L)	11.19 ± 3.99	10.78 ± 5.16	0.611	11.91 ± 5.74	10.10 ± 5.75	0.176	
Basal LH (IU/L)	3.75 ± 1.43	3.41 ± 1.82	0.235	3.40 ± 1.96	3.24 ± 2.16	0.728	
Basal estradiol (pg/ml)	53.03 ± 26.33	49.60 ± 24.80	0.417	40.42 ± 14.62	43.25 ± 22.89	0.550	
AFC	6.17 ± 3.36	6.25 ± 2.74	0.872	5.86 ± 2.88	6.92 ± 3.38	0.156	
AMH (ng/ml)	0.70 ± 0.35	0.67 ± 0.33	0.577	0.55 ± 0.29	0.60 ± 0.35	0.554	
Gn total dose (IU)	2,489.33 ± 860.00	2,352.42 ± 738.00	0.291	2,535.86 ± 816.30	2,457.26 ± 979.05	0.714	
Gn duration (days)	9.67 ± 2.33	9.32 ± 2.30	0.358	9.31 ± 2.29	9.47 ± 2.83	0.793	
Trigger day							
Estradiol (pg/ml)	1,384.42 ± 811.90	1,410.55 ± 745.00	0.838	1,090.43 ± 736.75	1,020.75 ± 686.40	0.670	
Progesterone (ng/ml)	0.64 ± 0.37	0.70 ± 0.60	0.490	0.56 ± 0.26	0.76 ± 1.09	0.327	
LH (IU/L)	2.89 ± 3.67	2.29 ± 1.73	0.267	2.21 ± 1.56	1.84 ± 1.38	0.273	

Dates were shown as mean ± SD or n (%).

FSH, follicle-stimulating hormone; LH, luteinizing hormone.

The comparison of laboratory parameters and pregnancy outcomes of the different MPA dose groups in normal BMI and high BMI patients is shown in **Table 2**. For normal BMI patients, the embryo implantation (33.78% vs. 18.97%, *P* = 0.012) was higher in the 8-mg/d MPA group than in the 10-mg/d MPA group. For high BMI patients, the HCG positive rate (55.00% vs. 25.00%, *P* = 0.028), clinical pregnancy rate (50.00% vs. 20.00%, *P* = 0.025), and cumulative pregnancy rate (37.74% vs. 13.79%, *P* = 0.023) were higher in the 10-mg/d MPA group than in the 8-mg/d MPA group. There were no statistically significant differences in the other indexes between the 8-mg/d MPA group and the 10-mg/d MPA group in both normal BMI and high BMI patients.

**Table 2:** Laboratory parameters and pregnancy outcomes of the different MPA dose groups.

Variable	Normal BMI			High BMI			
	8-mg/d MPA group (*n* = 52 cycles)	10-mg/d MPA group (*n* = 119 cycles)	*P-*value	8-mg/d MPA group (*n* = 29 cycles)	10-mg/d MPA group (*n* = 53 cycles)	*P-*value	
No. of oocytes retrieved	4.31 ± 3.20	3.87 ± 2.44	0.334	2.93 ± 2.15	3.25 ± 2.79	0.601	
No. of mature oocytes	3.73 ± 2.92	3.18 ± 2.25	0.185	2.59 ± 2.01	2.60 ± 2.26	0.972	
Mature oocyte rate (%)	85.57 ± 23.65	80.41 ± 30.64	0.234	72.32 ± 37.17	81.20 ± 36.40	0.301	
2PN fertilization rate (%)	58.84 ± 33.49	53.22 ± 37.30	0.352	50.82 ± 37.51	51.40 ± 39.33	0.949	
2PN cleavage rate (%)	83.97 ± 36.45	75.99 ± 41.90	0.211	65.52 ± 48.37	69.18 ± 46.16	0.736	
No. of high-quality embryos	1.50 ± 1.54	1.45 ± 1.53	0.857	1.07 ± 1.51	1.26 ± 1.33	0.547	
High-quality embryo rate (%)	49.57 ± 44.34	51.08 ± 43.42	0.835	44.02 ± 46.04	50.89 ± 44.44	0.511	
No. of transferable embryos	2.12 ± 1.84	1.90 ± 1.68	0.453	1.55 ± 1.48	1.58 ± 1.68	0.929	
Cycle cancellation rate, n (%)	17 (32.69)	38 (31.93)	0.922	13 (46.43)	20 (38.46)	0.531	
No. of FET cycles	41	97		20	40		
Type of embryos transferred, n (%)			0.226			0.677	
Cleavage- stage embryos	53 (71.62)	137 (78.74)		24 (75.00)	59 (78.67)		
Blastocysts	21 (28.38)	37 (21.26)		8 (25.00)	16 (21.33)		
HCG positive rate, n (%)	20 (48.78)	38 (39.18)	0.296	5 (25.00)	22 (55.00)	0.028	
Embryo implantation rate, n (%)	25 (33.78)	33 (18.97)	0.012	7 (21.88)	24 (32.00)	0.290	
Clinical pregnancy rate, n (%)	19 (46.34)	29 (29.90)	0.064	4 (20.00)	20 (50.00)	0.025	
Early miscarriage rate, n (%)	1 (5.26)	4 (13.79)	0.643	1 (25.00)	8 (40.00)	1.000	
Cumulative pregnancy rate, n (%)	19 (36.54)	27 (22.69)	0.060	4 (13.79)	20 (37.74)	0.023	
Cumulative live birth rate, n (%)	16 (30.19)	25 (21.01)	0.169	3 (10.34)	11 (20.75)	0.373	

Dates were shown as mean ± SD or n (%).

FET, frozen embryo transfer.

Multivariate logistic regression was used to analyze the effects of MPA dose, age, BMI, AFC, and the number of oocytes retrieved and mature oocytes on cumulative pregnancy and cumulative live birth. As shown by the results in **Table 3**, AFC and the number of mature oocytes were positively correlated with cumulative pregnancy (OR = 1.166, 95% CI: 1.010~1.347, *P* = 0.036; OR = 1.565, 95% CI: 1.066~2.300, *P* = 0.022) and cumulative live birth (OR = 1.169, 95% CI: 1.006~1.359, *P* = 0.041; OR = 1.500, 95% CI: 1.014~2.219, *P* = 0.042) in normal BMI patients, but there was no significant correlation between MPA dose and pregnancy outcome. In patients with high BMI, the dose of MPA was significantly correlated with cumulative pregnancy (OR = 0.199, 95% CI: 0.046~0.861, *P* = 0.031) but had no significant effect on cumulative live birth.

**Table 3:** Multivariate logistic regression analysis of pregnancy outcomes.

Pregnancy outcome	Variable	Normal BMI		High BMI	
		OR [95% CI]	*P*	OR [95% CI]	*P*
Cumulative pregnancy	8 mg/d vs. 10 mg/d MPA	1.914 [0.852–4.300]	0.116	0.199 [0.046–0.861]	0.031
	Age	0.956 [0.879–1.040]	0.299	0.954 [0.842–1.081]	0.461
	BMI	0.919 [0.720–1.173]	0.499	1.071 [0.780–1.471]	0.672
	AFC	1.166 [1.010–1.347]	0.036	1.212 [0.975–1.507]	0.083
	No. of oocytes retrieved	0.655 [0.655–1.284]	0.614	0.928 [0.616–1.702]	0.928
	No. of mature oocytes	1.565 [1.066–2.300]	0.022	1.527 [0.804–2.899]	0.196
Cumulative live birth	8 mg/d vs. 10 mg/d MPA	1.642 [0.705–3.823]	0.250	0.569 [0.124–2.611]	0.468
	Age	0.950 [0.870–1.038]	0.254	0.889 [0.762–1.037]	0.135
	BMI	1.113 [0.860–1.441]	0.415	1.045 [0.733–1.488]	0.809
	AFC	1.169 [1.006–1.359]	0.041	1.114 [0.883–1.405]	0.364
	No. of oocytes retrieved	0.957 [0.673–1.361]	0.806	1.163 [0.681–1.988]	0.580
	No. of mature oocytes	1.500 [1.014–2.219]	0.042	1.151 [0.614–2.161]	0.661

## 4 Discussion

The growth and development of follicles is accompanied by an increase in estradiol concentration, and estradiol induces LH surge through positive feedback to promote follicle maturation and ovulation (16). Early LH surge is the main cause of unexpected ovulation and cycle cancellation during COH, which is usually inhibited by GnRH agonists and GnRH antagonists (17). The PPOS protocol was first proposed in 2015 and was proven more effective in inhibiting premature LH surge by using exogenous progesterone than the GnRH antagonist protocol (18, 19). The theoretical basis of the PPOS protocol is that exogenous progesterone inhibits the appearance of LH surge by blocking the positive feedback of estradiol (16). In our study, patients in the different MPA dose groups did not have an early LH surge, which also suggested the effectiveness of the PPOS protocol in suppressing LH surge. However, due to the effect of progestogen on endometrial receptivity, all the embryos obtained need to be frozen (20). With the development of embryo freezing and warming technology, FET has been widely utilized. Consequently, the clinical application of the PPOS protocol has been further improved. It has been confirmed in subsequent studies that the PPOS protocol can improve pregnancy outcomes of POR patients (21–23). In addition, the PPOS protocol has the advantages of low cost, oral administration, and easy availability, making it a more economical and convenient option for POR patients.

At present, the pathogenesis of POR has not been fully elucidated, and it is difficult to formulate clinical management strategies. The 2011 Bologna criteria were the first criteria to standardize the definition of POR patients (1). However, the Bologna criteria lack age-stratified management, cannot sufficiently address the problem of population heterogeneity, and have limited predictive power for pregnancy outcomes (24). To better manage POR patients, the 2016 POSEIDON criteria classified POR patients into POSEIDON groups 1 and 2 due to abnormal ovarian response to exogenous gonadotropin and POSEIDON groups 3 and 4 due to decreased ovarian reserve based on age and the ovarian reserve parameters AFC and AMH, which improved the homogeneity and comparability of clinical studies and was conducive to providing more accurate assisted pregnancy strategies for POR patients (10, 25). Female age, BMI, AFC, and COH protocols were independent predictors of live birth in POR patients according to the POSEIDON criteria (26, 27). The previous retrospective analysis on the impact of BMI on the pregnancy outcome of FET showed that low BMI had no significant impact on the live birth rate, while obesity was closely associated with decreased clinical pregnancy rate and live birth rate (28, 29).

Our study first evaluated the effect of different MPA doses on clinical outcomes of POSEIDON group 3 and 4 patients with different BMI levels and found that the higher dose of MPA (10 mg/d) was associated with higher cumulative pregnancy rates in high BMI patients. Our results show that the 8-mg/d MPA group has a higher embryo implantation rate than the 10-mg/d MPA group in normal BMI patients, while the 10-mg/d MPA group has a higher HCG positive rate, clinical pregnancy rate, and cumulative pregnancy rate than the 8-mg/d MPA group in high BMI patients. The CLBR had a similar trend, but the difference was not statistically significant in normal BMI and high BMI patients, probably because of insufficient sample size. The results of multivariate logistic regression suggest a significant correlation between MPA dose and cumulative pregnancy in patients with high BMI. Our findings confirm that a sufficiently large dose of MPA (10 mg/d) is beneficial for cumulative pregnancy in patients with high BMI, although MPA (8 mg/d) is also enough to inhibit early LH peaks. Previous research showed that 10 mg/d of MPA and 4 mg/d of MPA had similar pregnancy and live birth outcomes in patients with normal ovarian reserve or normal BMI (18.5–25 kg/m^2^) (7). However, using 10 mg/d of MPA in infertile women with high BMI (≥25 kg/m^2^) resulted in higher embryo implantation rate, clinical pregnancy rate, and live birth rate rather than using 4 mg/d of MPA, possibly because the high MPA dose rescued physiological progesterone deficiency in patients with high BMI, which was partially consistent with our research results (8). Contrary to our expectations, there was no significant difference in the total dose and duration of gonadotropin between the 8-mg/d MPA group and the 10-mg/d MPA group, which was different from previous research results (7, 8). This difference may be due to the different populations included, different MPA doses, and BMI classification criteria in the studies.

Previous studies showed that obese patients often had poor IVF outcomes related to oocyte quality (8, 30). A single-center cohort study showed that embryo quality parameters in high BMI patients were not age-related but correlated with fatty acids in follicular fluid (31). A randomized controlled trial demonstrated that using MPA in the PPOS protocol can increase lipid levels in follicular fluid, promoting follicular development and oocyte maturation (32). This may be the reason why the clinical pregnancy rate of patients with high BMI in the 10-mg/d MPA group is better than that in the 8-mg/d MPA group. Interestingly, in our study, the mature oocyte rate and high-quality embryo rate in the 10-mg/d MPA group showed an increasing trend compared with the 8-mg/d MPA group, but there was no significant statistical difference, possibly due to the small sample size. In addition, due to the fact that current embryo evaluations are based on morphological scores, there are subjective biases and individual differences. Although there is no difference in embryo grading between the groups, different doses of MPA may affect the developmental potential of embryos through potential epigenetic effects. The specific reasons and possible mechanisms still need further investigation. In the future, we will focus on the individualized treatment of POR patients to find the optimal dosage of MPA in the PPOS protocol, in order to increase the number of oocytes obtained and improve the quality of oocytes while effectively suppressing the early LH surge.

This study had some limitations. First, the retrospective study has selective bias. Second, our research sample size was small. Third, although the baseline data between the different MPA dose groups were comparable, many confounding factors existed during the ovulation induction cycle and FET cycle. Therefore, large-sample prospective trials or multicenter randomized controlled trials are needed for further studies in the future.

In conclusion, our study confirmed for the first time that in POR patients, high BMI patients who used 10 mg/d of MPA had better cumulative pregnancy rate than those who used 8 mg/d of MPA, although there is no significant difference in cumulative live birth rate. In normal BMI patients, the 8-mg/d MPA group had a higher embryo implantation rate than the 10-mg/d MPA group, while cumulative pregnancy and cumulative live birth rates were similar. The impact of MPA on patient pregnancy outcomes still needs further prospective trials with a large sample size to verify.

## Data availability statement

The raw data supporting the conclusions of this article will be made available by the authors, without undue reservation.

## Ethics statement

The studies involving humans were approved by the Ethics Committee of Renmin Hospital of Wuhan University. The studies were conducted in accordance with the local legislation and institutional requirements. The participants provided their written informed consent to participate in this study.

## Author contributions

QZ: Data curation, Writing – original draft, Writing – review & editing. SH: Data curation, Writing – original draft. YM: Writing – original draft. TY: Conceptualization, Writing – review & editing. LM: Methodology, Writing – review & editing. JY: Supervision, Writing – review & editing. SL: Conceptualization, Funding acquisition, Methodology, Supervision, Writing – review & editing.
